# Improved ligand geometries in crystallographic refinement using *AFITT* in *PHENIX*

**DOI:** 10.1107/S2059798316012225

**Published:** 2016-08-31

**Authors:** Pawel A. Janowski, Nigel W. Moriarty, Brian P. Kelley, David A. Case, Darrin M. York, Paul D. Adams, Gregory L. Warren

**Affiliations:** a BioMaPs Institute, Center for Integrative Proteomics Research, Rutgers University, Piscataway, NJ 08854, USA; b Department of Chemistry and Chemical Biology, Rutgers University, Piscataway, NJ 08854, USA; c Molecular Biophysics and Integrated Bioimaging, Lawrence Berkeley National Laboratory, Berkeley, CA 94720, USA; d Novartis Institutes for BioMedical Research Inc., Cambridge, MA 02139, USA; e Department of Bioengineering, University of California at Berkeley, Berkeley, CA 94720, USA; f OpenEye Scientific Software, Santa Fe, NM 87508, USA

**Keywords:** macromolecular refinement, ligands, geometry restraints, *PHENIX*, *AFITT*

## Abstract

Modern crystal structure refinement programs rely on geometry restraints to overcome the challenge of a low data-to-parameter ratio. While the classical Engh and Huber restraints work well for standard amino-acid residues, the chemical complexity of small-molecule ligands presents a particular challenge. Most current approaches either limit ligand restraints to those that can be readily described in the Crystallographic Information File (CIF) format, thus sacrificing chemical flexibility and energetic accuracy, or they employ protocols that substantially lengthen the refinement time, potentially hindering rapid automated refinement workflows. *PHENIX*–*AFITT* refinement uses a full molecular-mechanics force field for user-selected small-molecule ligands during refinement, eliminating the potentially difficult problem of finding or generating high-quality geometry restraints. It is fully integrated with a standard refinement protocol and requires practically no additional steps from the user, making it ideal for high-throughput workflows. *PHENIX*–*AFITT* refinements also handle multiple ligands in a single model, alternate conformations and covalently bound ligands. Here, the results of combining *AFITT* and the *PHENIX* software suite on a data set of 189 protein–ligand PDB structures are presented. Refinements using *PHENIX*–*AFITT* significantly reduce ligand conformational energy and lead to improved geometries without detriment to the fit to the experimental data. For the data presented, *PHENIX*–*AFITT* refinements result in more chemically accurate models for small-molecule ligands.

## Abstract 2

A method for the more accurate refinement of small molecules and ligands in biomolecular structures is provided. Improved ligand geometry is obtained *via* an all-atom molecular-mechanics force field.

## 1. Introduction   

Structural knowledge is fundamental to our understanding of biomolecular function in drug-discovery and drug-optimization efforts. X-ray crystallography remains the pre-eminent method for obtaining detailed structural information about molecules. Continued advances in data collection and processing, model building and structure refinement have gone a long way towards making crystallography a semi-automated, reliable technique for high-throughput structural biology. In the course of crystallographic structure solution, the process of refinement is used to optimize the atomic coordinates against the experimental data. However, because of the low data-to-parameter ratio in a typical experiment, additional *a priori* knowledge must be introduced into the optimization algorithm to make reliable model generation tractable. Usually, this additional knowledge is in the form of stereochemical restraints for bond lengths and angles and steric exclusions as well as additional restraints for dihedrals, chirality and other geometry restraints.

For standard biomolecular residues (proteins and nucleic acids), most modern refinement programs base these restraints on the so-called Engh and Huber restraints developed in 1991 (Engh & Huber, 1991 ▸) from a survey of small-molecule crystal structures and with later corrections added in 1996 for nucleic acids (Parkinson *et al.*, 1996 ▸) and in 2001 (Engh & Huber, 2001 ▸) for amino-acid residues. Engh and Huber restraints function reasonably well for standard residues, but even in this case deficiencies have been exemplified (Davis *et al.*, 2003 ▸; Moriarty *et al.*, 2014 ▸; Priestle, 2003 ▸; Touw & Vriend, 2010 ▸). On the other hand, modeling of small-molecular ligands presents a particular challenge owing to their more complex chemistry, conformations and energetics. Thus, small-molecule ligands can be inaccurately modeled by the standard set of restraints (Davis *et al.*, 2003 ▸; Kleywegt *et al.*, 2003 ▸; Kleywegt & Jones, 1998 ▸; Moriarty *et al.*, 2014 ▸; Priestle, 2003 ▸; Touw & Vriend, 2010 ▸). In fact, recent studies suggest that as many as 60% of the structures deposited in the Protein Data Bank (PDB; Berman *et al.*, 2000 ▸, 2003 ▸) may contain questionable ligand conformations (Liebeschuetz *et al.*, 2012 ▸; Pozharski *et al.*, 2013 ▸).

Significant effort has been placed into developing tools for the accurate generation of ligand restraints in crystallographic models. Some (Davis *et al.*, 2003 ▸; Lebedev *et al.*, 2012 ▸; Moriarty *et al.*, 2009 ▸; Schüttelkopf & van Aalten, 2004 ▸; Smart *et al.*, 2012 ▸; Wlodek *et al.*, 2006 ▸) employ sophisticated approaches to derive the same type of stereochemical restraints as those used by Engh and Huber for standard residues. In other words, these restraints must conform to the standard Crystallographic Information File (CIF) restraint dictionary format provided to the restraint program (Brown & McMahon, 2002 ▸; Hall *et al.*, 1991 ▸, Vagin *et al.*, 2004 ▸). Other approaches focus on more accurate ligand representation through the use of more elaborate protocols using force fields, semi-empirical or quantum methods.

The former approaches suffer from a potentially too simplistic representation of the ligand by using the restraint categories found in the standard restraint CIF format that insufficiently models or wholly ignores energetic effects such as electrostatics and dispersion forces. The latter approaches are often complicated to use, requiring multiple additional steps from the user. They are therefore difficult to integrate into an automated workflow and the speed requirements of many modern-day high-throughput laboratories such as those involved in pharmaceutical drug discovery (Borbulevych *et al.*, 2016 ▸; Borbulevych, Plumley *et al.*, 2014 ▸).

We present a more accurate but efficient structural modeling of small molecules in the refinement process using the combined power of two crystallographic applications. *PHENIX* (Adams *et al.*, 2010 ▸) is the widely popular suite of software for integrated crystallography that includes the *phenix.refine* (Afonine *et al.*, 2012 ▸) application for refinement; *AFITT* (v.2.4.0.4; http://www.eyesopen.com/afitt; Wlodek *et al.*, 2006 ▸) is OpenEye’s package for automated ligand placement in real-space density. *AFITT* models ligand conformation and stereochemistry with the well regarded Merck Molecular Mechanics Force Field (MMFF; Halgren, 1996*a* ▸,*b* ▸,*c* ▸,*d* ▸, 1999 ▸; Halgren & Nachbar, 1996 ▸), but until now has written MMFF-derived restraints to the standard CIF format restraint files, sometimes to the detriment of the ligand conformation. By seamlessly integrating AFITT with *PHENIX*, the user gains the powerful advantage of a full molecular-mechanics representation of the ligand while being able to maintain the same efficient refinement workflow. Furthermore, alternating steps of standard macromolecular refinement followed by highly accurate ligand refinement is no longer necessary as both sets of restraints are applied simultaneously.

Here, we provide a comparison of refinements on a test set of 189 ligands and 304 ligand instances. We have chosen to only compare refinement using *AFITT*-derived CIF restraint dictionaries *versus* obtaining the ligand geometry gradients in refinement directly from *AFITT* (*PHENIX*–*AFITT*). Thus, our comparison is not complicated by differences in potential functions and target values for the methods used by various other ligand restraint-file generation and modeling methods (Borbulevych, Moriarty * et al.*, 2014 ▸; Borbulevych, Plumley*, et al.*, 2014 ▸; Davis *et al.*, 2003 ▸; Fu *et al.*, 2011 ▸; Lebedev *et al.*, 2012 ▸; Moriarty *et al.*, 2009 ▸; Schüttelkopf & van Aalten, 2004 ▸; Smart *et al.*, 2010 ▸, 2012 ▸; Yu *et al.*, 2005 ▸). Instead, differences hinge only on the improvement gained by representing the ligand with the full molecular-mechanics force field during the course of refinement. *PHENIX*–*AFITT* refinements yield the expected improved lower-energy small-molecule structures while maintaining the same degree of agreement with experiment. Thus, a *PHENIX*–*AFITT* refinement provides the user with a fully integrated ligand refinement that ensures accurate modeling of ligand conformation and chemistry. The implementation of *AFITT* in *PHENIX* is versatile, easy to use and powerful. Refinements can include different types of ligands and multiple instances of each ligand type. Support for ligands with full or partial alternate conformations is fully integrated, as is refinement of ligands covalently bound to the macromolecule.

## 2. Methods   

*Phenix.refine* (Afonine *et al.*, 2012 ▸) optimizes a crystal structure *via* a series of repeated cycles. During each cycle a series of parameters of the user’s choosing are optimized. These usually include the atomic coordinates and the isotropic atomic displacement parameters, but can also include, for example, translation–libration–screw parameters, bulk-solvent scaling and anisotropic atomic displacement parameters. Each optimization is conducted by minimizing a residual function of the model against the experimental data using a maximum-likelihood approach.

*AFITT* (Wlodek *et al.*, 2006 ▸) is a package developed by OpenEye Scientific Software for small-molecule real-space fitting in biomolecular crystallography. It uses a combination of a real-space electron-density shape-matching algorithm and a molecular-mechanics force field to fit small ligands into density while maintaining accurate chemical geometry. *AFITT* uses an ‘adiabatic’ method to find the best relative weight between these two components. It can be run without a solvent model or using either the Sheffield (Grant *et al.*, 2007 ▸) or the Poisson–Boltzmann scheme (Grant *et al.*, 2001 ▸) to model solvation effects. *AFITT* uses the Merck Molecular Mechanics Force Field (MMFF94; Halgren, 1996*a* ▸,*b* ▸,*c* ▸,*d* ▸, 1999 ▸; Halgren & Nachbar, 1996 ▸). This force field was designed to reproduce *ab initio* accuracy in a broad range of chemical functionality and has been shown to produce satisfactory results with the small molecules typically encountered in biomolecular crystallography (Fu *et al.*, 2011 ▸; Gundertofte *et al.*, 1996 ▸; Halgren, 1999 ▸).

In the case of reciprocal-space atomic coordinate refinement, the *PHENIX* refinement target function has the form
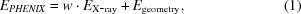
where *E*_X-ray_ is the residual of the structure factors, *E*_geometry_ is the residual owing to the Engh and Huber restraints (Engh & Huber, 1991 ▸, 2001 ▸) and *w* is a weighting factor. If a ligand is present, the *E*_geometry_ term can further be divided, 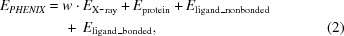
where *E*_ligand_bonded_ represents the so-called bonded terms in the geometry restraints that include bonds, angles, torsion angles, chiral and planar restraints. *E*_ligand_nonbonded_ represents the nonbonded terms that in the case of Engh and Huber are the atomic steric overlap restraints. For the *AFITT*-generated CIF restraints the bond and angle target values are the MMFF94 target values. The torsion-angle values are taken from the ligand conformation and periodicity assigned by analysis of the MMFF94 functional for each torsion. On the other hand, during the *PHENIX*–*AFITT* refinement introduced here, the last term in (3) is replaced by a residual calculated by *AFITT*, 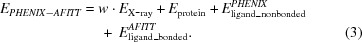
The implementation in *PHENIX* combines the *phenix.refine* refinement scheme and optimization algorithm while using *AFITT* to obtain the *E*_ligand_bonded_ part of the residual. The *AFITT* *E*_ligand_bonded_ gradient consists of stretch, stretch–bend, bend, torsion, out-of-plane, van der Waals and Coulomb terms. The *AFITT* gradient is applied only to ligands specified by the user. The current implementation does not take into account protein–ligand interaction terms other than steric overlap. It is important to note that there are no equivalent terms in the geometry restraint CIF file method for attractive van der Waals (present in the van der Waals term ) or electrostatics (present in the Coulomb term).

A *PHENIX*–*AFITT* refinement is invoked with phenix.refine mymodel.pdb mymodel.mtz myligand.cif use_afitt=True afitt.ligand_file=myligand.cif afitt.ligand_names=BCL.

This implementation automatically searches for all instances of the ligand specified by the user (bacteriochlorophyll A in the example above) and uses *AFITT* to calculate the geometry gradients for these instances. More than one type of ligand can be included. The required restraints CIF dictionary file specifies the required atom type and bond topology for *AFITT*, but not the actual restraint force constants, which are calculated internally by *AFITT*. This implementation also accounts for alternate conformations of ligand atoms and ligands covalently bound to the macromolecule. A heuristically determined weight, based on *R*_free_ and ligand energy, of 10 is placed on the *E* ^*AFITT*^_ligand_bonded_ term by default, but the user also has the option of modifying this weight. Additionally, there is a simple command-line tool to quickly obtain MMFF ligand energies from a given PDB coordinate file.

The root-mean-square deviation (r.m.s.d.) values reported were calculated using OpenEye’s r.m.s.d. (OERMSD) method found in *OEChem* (v.2015.October; http://www.eyesopen.com/oechem-tk). All r.m.s.d. values were calculated for heavy atoms only, with no ligand overlay, and accounting for automorphisms, which is a form of chemical symmetry that takes into account the atoms in the ligand that can be relabelled without changing the chemical structure of the ligand, *e.g.* carboxylate O atoms are equivalent. The Python script used to calculate the values (rmsd.py) is included along with all of the other data for download at http://www.phenix-online.org/phenix_data/Phenix_AFITT/.

## 3. Results   

Testing of the implementation of *AFITT* in *PHENIX* was performed using a set of 189 protein–ligand PDB structures taken from the Iridium data set (Warren *et al.*, 2012 ▸). This set contains a chemically varied and largely drug-like set of ligands. While this data set is an annotated and curated set, we have chosen to use structures that were annotated as not being highly reliable. Some structures contained multiple small molecules and/or multiple instances, resulting in 304 small-molecule instances in total. Because many of the structures lacked reflection test sets (to calculate *R*_free_; Brünger, 1993 ▸), a new test set of reflections was assigned and each model was refined using *phenix.refine* with the default strategy for ten macrocycles to remove ‘memory’ of the original test set. Next, each structure was refined for a further five macrocycles using the default strategy and with the ligand modeled either using an *AFITT*-derived CIF dictionary file (hereafter referred to as ‘*AFITT*–CIF’ refinement) or with the new *PHENIX*–*AFITT* implementation. This focused strategy, as opposed to comparing results with another non-*AFITT* ligand-restraint tool, allowed rigorous testing of the benefits of implementing ligand optimization in the way advocated here; that is, by applying a full molecular-mechanics treatment of the ligand but in a manner fully integrated with the refinement optimizer.

**Figure 1:**
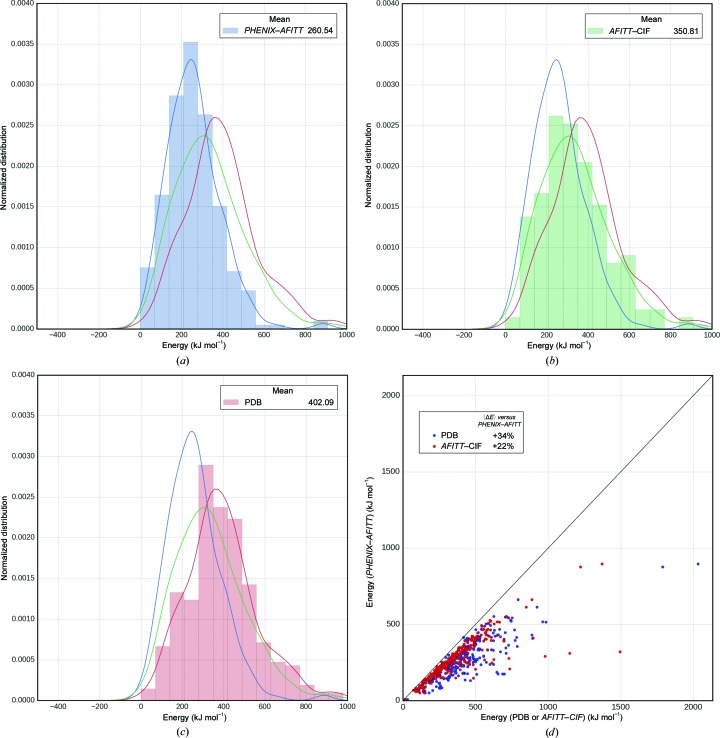
Ligand conformational energies from PDB-deposited models, *AFITT*–CIF refinement and *PHENIX*–*AFITT* refinement. (*a*, *b*, *c*) Histograms for *PHENIX*–*AFITT* (*a*), *AFITT*–CIF (*b*) and PDB-deposited (*c*) energies with kernel density estimates (KDE) of the distributions for the full set of test ligand energies. Means of each set of ligand conformation energies are shown in the legend. (*d*) A scatter plot comparing the conformation energy of each ligand obtained from a *PHENIX*–*AFITT* refinement against either the deposited PDB model (blue dots) or the models after refinement with an MMFF-derived CIF dictionary file (red dots). The mean percentage reduction in energy from using the *PHENIX*–*AFITT* protocol is 34% *versus* the PDB conformations and 22% *versus* *AFITT*–CIF.

The *PHENIX*–*AFITT* method is expected to produce ligand conformations with lower MMFF94 ligand energies as MMFF94 is part of the target function. *PHENIX*–*AFITT* refinement produces ligand models with significantly lower ligand energies, both compared with the original deposited coordinates and with refinement using an MMFF-based CIF restraints file (Fig. 1 ▸). For our test set, the mean post-refinement ligand energy was 402.1 ± 210.1 kJ mol^−1^ for the set of deposited structures, 350.8 ± 199.9 kJ mol^−1^ for the *AFITT*–CIF refined structures and 260.5 ± 128.4 kJ mol^−1^ for the *PHENIX*–*AFITT* refined structures. This signifies an average reduction of ligand conformational energies of 34% *versus* the deposited conformation in the PDB and 22% *versus* refinement using MMFF-derived CIF restraint dictionaries. Fig. 1 ▸(*d*) shows scatter plots of the *PHENIX*–*AFITT* refined ligand energies *versus* either the deposited PDB or *AFITT*–CIF refined energies on a per-model basis. It is apparent that *PHENIX*–*AFITT* refinement results on average in models with a significant and in most cases a substantial reduction of ligand conformational energy. Statistical significance was measured using a dependent paired t-test (rel t-test; Student, 1908 ▸) and a two-sample distribution Kolmogorov–Smirnov test (ks_2samp; Kolmogorov, 1933 ▸; Pearson & Hartley, 1972 ▸; Smirnov, 1948 ▸). The Cohen *d* (Cohen, 1988 ▸) was used to test whether the difference of the means was substantial. For PDB-deposited *versus* *PHENIX*–*AFITT* the rel t-test *p* was 10^−57^, the ks_2samp *p* was 10^−25^ and the Cohen *d* was 0.81 or large. Thus, when comparing the MMFF energy of ligand conformations from *PHENIX*–*AFITT* refinements and deposited structures, *PHENIX*–*AFITT* generates both significantly and substantially lower energy conformations. For *PHENIX*–*AFITT* *versus* *AFITT*–CIF the rel t-test *p* was 10^−33^, the ks_2samp *p* was 10^−9^ and the Cohen *d* was 0.54 or moderate. While *PHENIX*–*AFITT* generates significantly lower energy conformations *versus* *AFITT*–CIF, the difference is only moderate in size. Last, the greatest energy reduction using *AFITT* in *PHENIX* (the distance below the identity line) tends to occur in the ligands with highest starting energies. In other words, the higher the initial conformational energy the more a *PHENIX*–*AFITT* refinement is able to reduce it.

**Figure 2:**
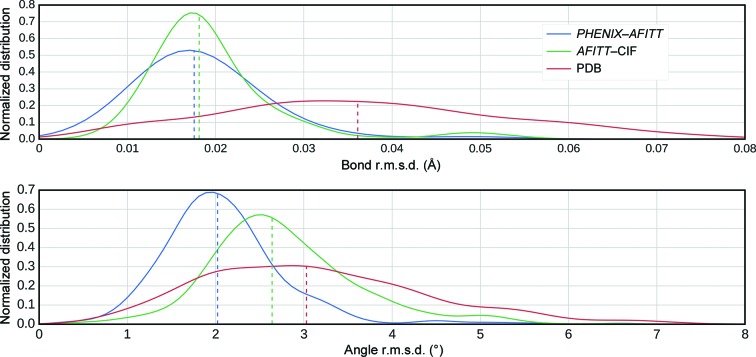
*Mogul* validation of the PDB-deposited, *AFITT*–CIF refined and *PHENIX*–*AFITT* refined ligand conformations. The top panel shows the bond r.m.s.d. distribution in Å and the bottom panel shows the angle r.m.s.d. distribution in degrees. R.m.s.d. is relative to the *Mogul* library of ‘ideal’ bonds and angles.

To further validate refinement quality, the *Mogul* software (Bruno *et al.*, 2004 ▸) was used to assess the post-refinement ligand geometries. *Mogul* is a knowledge-based library derived from experimental small-molecule crystal geometries in the Cambridge Structural Database (CSD; Allen, 2002 ▸). *Mogul* (Bruno *et al.*, 2004 ▸) has been shown to be an excellent independent evaluator of computationally derived geometries. The results (Fig. 2 ▸) show that both the *PHENIX*–*AFITT* refined and the *AFITT*–CIF refined geometries are significantly better than those found in the PDB and that *PHENIX*–*AFITT* geometries are better than *AFITT*–CIF geometries. The respective mean r.m.s.d. scores for bonds were 0.036 ± 0.017 Å for the deposited PDB set, 0.019 ± 0.007 Å for *AFITT*–CIF and 0.018 ± 0.016 Å for the *PHENIX*–*AFITT* protocol. For bond lengths, using a two-sample *z*-test, both *PHENIX*–*AFITT* and *AFITT*–CIF have significantly lower deviations from the CSD, with *p *= 0.0005 and *p* = 0.0009, respectively. The difference is substantial in both cases as measured by the Cohen *d*, where *d* = 1.6 (large) for deposited *versus* *AFITT*–CIF and *d* = 1.1 (large) for deposited *versus* *PHENIX*–*AFITT*. There is no statistically significant difference in the means between *PHENIX*–*AFITT* and *AFITT*–CIF as measured by the two-sample *z*-test (*p* = 0.39) and the Cohen *d* of 0.08 (trivial). However, if difference in the distribution is tested using ks_2samp there is a statistically significant difference in the distributions (*p* = 10^−6^) and this difference is visualized in the top panel of Fig. 2 ▸, where there is a large shoulder towards lower deviation for the *PHENIX*–*AFITT* curve. For angles, the respective mean difference was 3.11 ± 1.25° for the deposited PDB set, 2.77 ± 0.84° for *AFITT*–CIF and 2.09 ± 0.72° using *PHENIX*–*AFITT*. This is a statistically significant and in most cases substantial reduction in deviation, with *z*-test *p* values of *p* = 10^−13^ (Cohen *d* = 0.4 or moderate) for deposited *versus* *AFITT*–CIF, *z*-test *p* = 10^−100^ (Cohen *d* = 1.1 or large) for deposited *versus* *PHENIX*–*AFITT* and *z*-test *p* = 10^−122^ (Cohen *d* = 0.9 or large and ks_2samp *p* = 10^−98^) for *AFITT*–CIF *versus* *PHENIX*–*AFITT*. These data clearly show that *PHENIX*–*AFITT* refinements generate models with not only reduced conformational energies, as assessed by the MMFF force field, but more accurate bonds and angles when compared with small-molecule crystal geometries.

**Figure 3:**
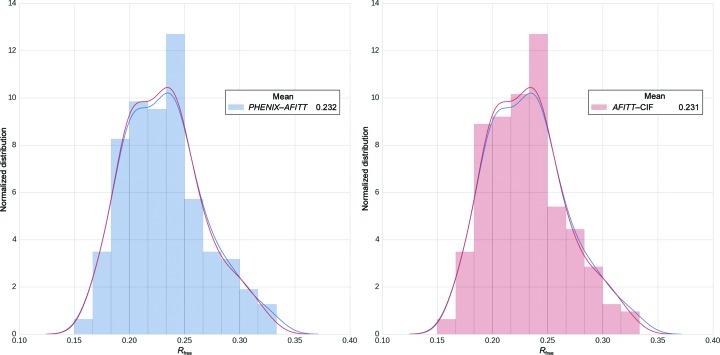
*R*_free_ distributions and histograms after refining the test set using either *AFITT*–CIF (right) or the *PHENIX*–*AFITT* (left) protocol. Means of each distribution are shown in the legend.

While significantly reducing ligand energies, *PHENIX*–*AFITT* refinements did not result in poorer agreement with experimental data both at the global and at the individual ligand-residue level. No differences in global fit measures such as *R*_free_ were expected. The mean *R*_free_ of the structures refined with *AFITT*–CIF was 0.231 ± 0.035, while for those refined with the *PHENIX*–*AFITT* protocol the mean *R*_free_ was 0.232 ± 0.036 (Fig. 3 ▸). A pairwise comparison of *PHENIX*–*AFITT* and *AFITT*–CIF refinements yields a mean difference in *R*_free_ between the two methods of 0.0012, which is a statistically significant difference as measured by a rel t-test *p* of 10^−9^, but is not significant or substantial as measured by a ks_2samp *p* of 0.995 or a Cohen *d* of 0.03 or trivial. Thus, on average *AFITT*–CIF results in a slightly lower *R*_free_, but this difference is very small (0.0012 on average). We found only one case with an *R*_free_ difference greater than 0.01. This was the case of PDB entry 1ctr, which resulted in a Δ*R*_free_ of 0.025. However, the starting model for PDB entry 1ctr is problematic in itself with 32% rotamer outliers, a clashscore of 50 and a *MolProbity* (Chen *et al.*, 2010 ▸) score of 4.07. In addition, all low-resolution data beyond 6 Å are missing in the deposited structure factors. Thus, one might expect to see large *R*-factor fluctuations upon refinement using even slightly different protocols in this case.

**Figure 4:**
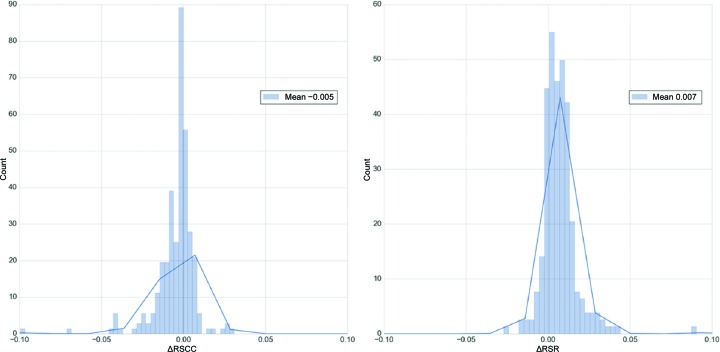
The difference distribution of the ligand RSCC (left) and RSR (right) for the *AFITT*–CIFF *versus* *PHENIX*–*AFITT* models. The mean difference for each distribution is shown in the legend.

Ligand local fit to electron density was measured using the real-space correlation coefficient (RSCC) and real-space *R* factor (RSR) (Jones *et al.*, 1991 ▸) calculated using the in-house program *DCC* (Yang *et al.*, 2016 ▸) kindly provided by Huanwang Yang from the Protein Data Bank. The mean RSCC for *AFITT*–CIF was 0.944 ± 0.040 and that for *PHENIX*–*AFITT* was 0.939 ± 0.047. The mean RSCC pairwise difference was −0.005 (see Fig. 4 ▸), which is a statistically significant difference as measured by a rel t-test (*p* = 10^−8^), but not significant or substantial as measured by ks_2samp (*p* = 0.53) and a Cohen *d* of 0.12 or trivial. The mean RSR for *AFITT*–CIF was 0.125 ± 0.050 and that for *PHENIX*–*AFITT* was 0.132 ± 0.055. The mean pairwise difference of 0.007 (see Fig. 4 ▸) was again a statistically significant difference as measured by the rel t-test *p* of 10^−17^ but was not significant or substantial as measured by the ks_2samp *p* of 0.11 and a Cohen *d* of 0.14 or trivial. Thus, *PHENIX*–*AFITT* generates conformations with significantly lower conformational strain energy but with an equivalent fit to the data. However, the consistent slightly poorer numerical fits of the *PHENIX*–*AFITT* models might be an indication that the heuristic used to assess the default of weight of 10 placed on the *E* *^AFITT^*_ligand_bonded_ gradient needs to be reassessed and a slightly lower default weight chosen.

**Figure 5:**
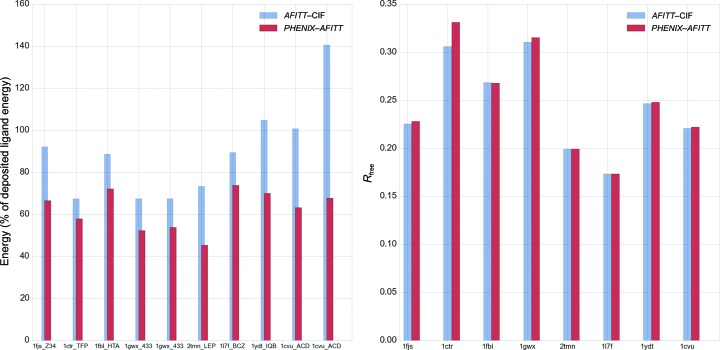
Comparison of eight randomly selected PDB structures. The left panel shows energies obtained with *AFITT*–CIF refined and *PHENIX*–*AFITT* refined ligand restraints as a percentage of the deposited ligand energy. Labels provide the PDB code followed by the three-letter code for the ligand. Some PDB structures have more than one instance of a ligand. The right panel shows the *R*_free_ obtained after refinement with Engh and Huber or *AFITT* geometry restraints on the ligands.

**Figure 6:**
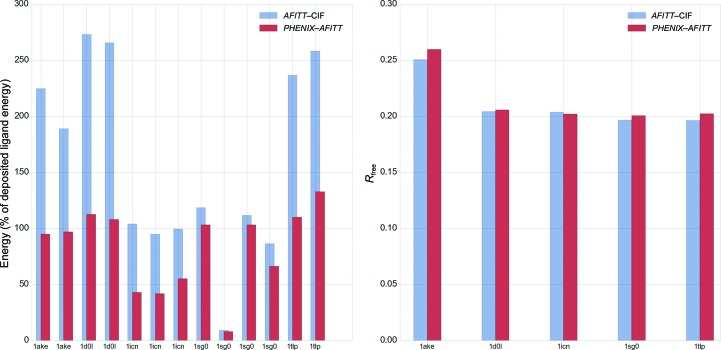
Comparison of five PDB structures containing ligand instances with alternate conformations. It is important to note that only for PDB entries 1ake and 1icn were the deposited ligand conformations modeled as alternate conformations. All labels and statistics are as in Fig. 4 ▸.

Fig. 5 ▸ shows a more detailed comparison of eight randomly selected structures from the test set with a total of ten ligands. As can be seen, *PHENIX*–*AFITT* refinement leads to significantly lower energies in all cases. In a few cases (for example the second ACD instance in PDB entry 1cvu) *AFITT*–CIF restraints lead to ligand energies that are much higher than even the deposited coordinates, while using *AFITT* directly in *PHENIX* gives the expected lower energies. At the same time, a comparison of *R*_free_, RSCC and RSR for the ligand shows that the fit to experimental data remains essentially the same between the two refinements. The structure with the highest *R*_free_ difference in the entire data set, PDB entry 1ctr discussed previously, was included in the panel. *PHENIX*–*AFITT* can also handle ligands with alternate conformations. Fig. 6 ▸ shows a similar comparison of energies and *R*_free_ factors for five PDB structures containing multiple ligands with alternate conformations. Analysis of these data for *AFITT*–CIF is complicated by the fact that only two of the deposited ligand conformations were modeled with alternate conformations (PDB entry 1ake copy 1 and 1icn). However, the conclusions for the *PHENIX*–*AFITT* models are similar in that these models have similar or substantially lower energy.

**Figure 7:**
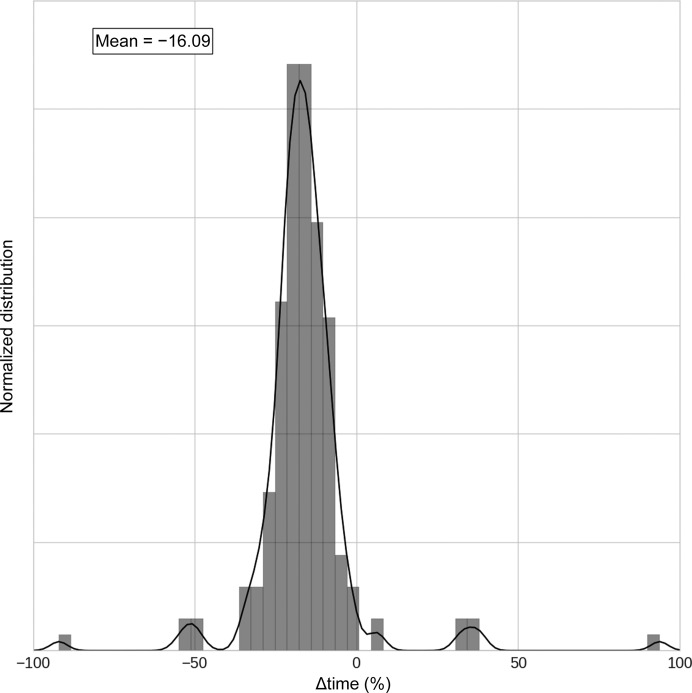
Difference in run time between traditional Engh and Huber and *PHENIX*–*AFITT* refinement as a percentage of the Engh and Huber refinement run time. Positive numbers indicate that the *PHENIX*–*AFITT* refinement is faster and negative numbers that the *PHENIX* Engh and Huber refinement is faster. Five outliers (PDB entries 1q41, 1sq5, 1q1g, 1hq2 and 1dd7) have been omitted from the plot.

Structure refinement with the *PHENIX*–*AFITT* protocol is somewhat slower than refinement with a previously prepared CIF dictionary file. Fig. 7 ▸ presents a histogram of runtime differences between *AFITT*–CIF and *PHENIX*–*AFITT* refinement as a percentage of the *AFITT*–CIF runtime. In general, a *PHENIX*–*AFITT* refinement is slower by an average of 16% compared with the same refinement using a dictionary file with Engh and Huber-style restraints. Subsequent analysis has shown that most of the computational cost comes from a license check that is being performed for each gradient calculation. In addition, there were five structures with refinement times that were more than twice as slow as with the traditional algorithm: 1q41 (2235%), 1sq5 (1141%), 1q1g (440%), 1hq2 (219%) and 1dd7 (110%). There is no clear pattern as to why the refinement time for these structures was longer. The ligands range in size from molecular weights of 195 to 480 with from zero to nine rotatable bonds. For 1q1g and 1sq5 there are multiple copies of the ligand (six and four, respectively) but 1q41 with the longest refinement time has only two copies and zero rotatable bonds. The cause of the increase in refinement time is still under investigation. These five outliers have been omitted from the plot.

**Figure 8:**
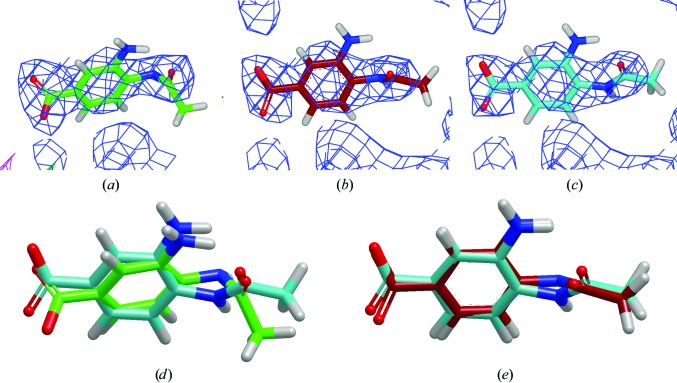
A depiction of the conformations of the second copy of the ligand from PDB entry 1ive. (*a*), (*b*) and (*c*) show the deposited (C atoms colored green), *AFITT*–CIF (C atoms coloured red) and *PHENIX*–*AFITT* (C atoms colored turquoise) conformations, respectively. The density shown is σ_A_-weighted 2*F*_o_ − *F*_c_ density contoured at 1σ and the difference map was contoured at 3σ. (*d*) shows the deposited and *PHENIX*–*AFITT* conformations using the previously described color scheme, where the r.ms.d. is 0.81 Å and the energy difference is 134 kJ mol^−1^. (*e*) shows an overlay of the *AFITT*–CIF and *PHENIX*–*AFITT* conformations. The r.m.s.d. is 0.31 Å and the energy difference is 41.6 kJ mol^−1^. There are no examples in this data set where the r.m.s.d. between the *AFITT*–CIF and *PHENIX*–*AFITT* conformations exceeds 0.4 Å, yet the energy difference between the two conformations was almost always large (>10 kJ mol^−1^).

**Figure 9:**
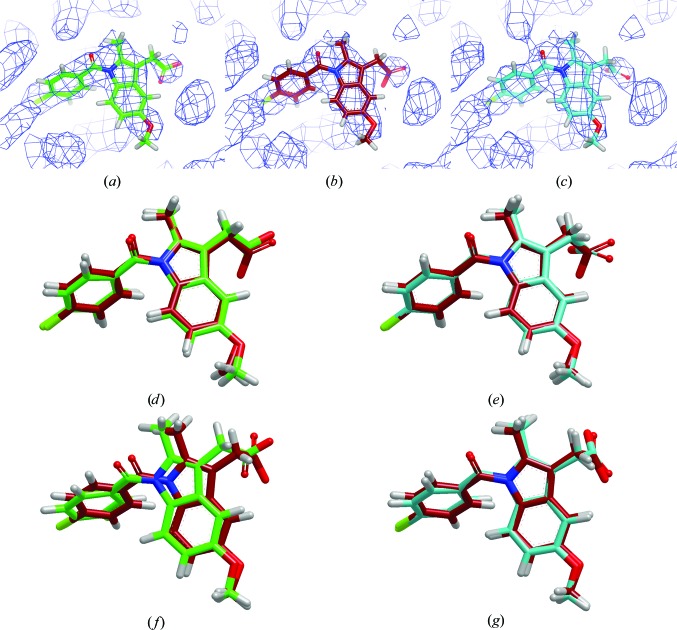
A depiction of the conformations of the first copy of the ligand from PDB entry 4cox. (*a*), (*b*) and (*c*) show the deposited (C atoms colored green), *AFITT*–CIF (C atoms colored red) and *PHENIX*–*AFITT* (C atoms colored turquoise) conformations, respectively. The density shown is σ_A_-weighted 2*F*_o_ − *F*_c_ density contoured at 1σ. No difference density was observed when the density was contoured at 3σ. There are a total of four copies of the ligand in this deposition. (*d*) and (*e*) show the overlay for the first copy between the deposited and *AFITT*–CIF and between the *AFITT*–CIF and *PHENIX*–*AFITT* conformations, respectively, using the previously described coloring scheme. The r.m.s.d. between the deposited and the *AFITT*–CIF conformation was 0.24 Å, whereas the difference between the *AFITT*–CIF and *PHENIX*–*AFITT* conformations was 0.38 Å. In this case the *AFITT*–CIF refinement was unable to find the low-energy conformation generated by the *PHENIX*–*AFITT* refinement, as shown by the r.m.s.d. and the difference energies of 1.57 kJ mol^−1^ for the deposited *versus* *AFITT*–CIF and 119 kJ mol^−1^ for the deposited *versus* *PHENIX*–*AFITT* conformation. (*f*, *g*) Copy 4 presents a different result. The *AFITT*–CIF and *PHENIX*–*AFITT* methods both find a lower energy conformation where the r.m.s.d. for the deposited *versus* *AFITT*–CIF and deposited *versus* *PHENIX*–*AFITT* was 0.7 Å, but the r.m.s.d. for the *AFITT*–CIF and *PHENIX*–*AFITT* conformations was only 0.20 Å. The energy differences were 31.2 kJ mol^−1^ for deposited *versus* *AFITT*–CIF and 117 kJ mol^−1^ for deposited *versus* *PHENIX*–*AFITT*. An interesting observation from this example is that the *PHENIX*–*AFITT* method appears to be more consistent at finding low-energy conformations (the standard deviation across the four samples was 4.08 kJ mol^−1^), whereas the *AFITT*–CIF method was not as consistent (standard deviation of 19.6 kJ mol^−1^).

The data presented until now have been aggregate data showing that *PHENIX*–*AFITT* reduces conformational energy on average while improving small-molecule geometry. This section will present two examples of the maximum change or difference in the coordinates observed for this data set. The coordinate differences between the *AFITT*–CIF and *PHENIX*–*AFITT* conformations were less than 0.3 Å for all but 22 cases out of the 304 in this data set. An example of one of the larger differences is shown in Fig. 8 ▸ for PDB entry 1ive. Both copies of this ligand have a root-mean-square deviation (r.m.s.d.) that is only slightly greater than 0.3 Å between the *AFITT*–CIF and *PHENIX*–*AFITT* conformations, but the difference in energy is greater than 40 kJ mol^−1^. Very small coordinate changes have a profound effect on the conformational energy of the ligand. There are no examples in this data set where the r.m.s.d. difference in the coordinates for the *AFITT*–CIF and *PHENIX*–*AFITT* conformations is greater than 0.4 Å, yet the difference in energy was almost always large (>10 kJ mol^−1^). The second example of PDB entry 4cox, shown in Fig. 9 ▸, highlights another difference in performance between *AFITT*–CIF and *PHENIX*–*AFITT*. All of the refinements in this study used the same starting coordinates: the deposited coordinates for the ligand. If both methods were equivalent they should produce similar results with a similar amount of variability. As illustrated in Fig. 9 ▸, this was not the case. *AFITT*–CIF refinements had a much larger variability in the energy, as measured by standard deviation, for the conformations generated for 4cox and 21 other cases (data not shown) where the number of copies of the ligand was greater than or equal to three. This result is particularly troubling. Even though the same CIF restraints file was used for the coordinates, it appears that *AFITT*–CIF refinements exhibit chaotic-like behavior (Feher & Williams, 2012*a* ▸,*b* ▸), where small changes in the coordinates result in large changes in energy, *versus* *PHENIX*–*AFITT* refinements. An alternative explanation is that the *PHENIX*–*AFITT* refinement method has a larger radius of convergence.

## 4. Discussion   

The *PHENIX*–*AFITT* protocol is a new tool that decreases ligand conformational energy *versus* the geometry restraints typically used in modern refinement programs. By implementing an interface to *AFITT* into *PHENIX* refinement, a more accurate set of geometric chemical gradients are made available, leading to a significant reduction in ligand conformational energies and a significant improvement in ligand geometries. This is accomplished without detriment to the fit of the model to experimental data and with only a modest increase in refinement time. *AFITT* is fully integrated with *phenix.refine*, is easy to use and automatically handles multiple ligands, alternate conformations and covalent linkage. A user’s guide is available online in the *PHENIX* documentation under the heading ‘Structure Refinement and Restraint Generation’.

The *PHENIX*–*AFITT* protocol not only improves on the deposited PDB ligand geometries but also on those obtained with refinements using a CIF-format restraints file derived from the same MMFF force field that *AFITT* uses. This is noteworthy because it underscores that improved refinement results are not solely the result of using better or more consistent dictionary file target values but also of how the force field is implemented within the refinement target function. Most target functions in use today only allow representation of bond, angle, simplified dihedral and atomic overlap penalty terms. Thus, a crystal structure refinement restraints representation of the geometry parameters of a ligand necessarily constrains the force field into a more primitive representation *versus* what is present in MMFF. For example, nonbonded interactions (electrostatics and van der Waals forces) are no longer accurately represented in the restraints format. This study cannot and does not prove that CIF-style restraints contain methodological flaws when attempting to accurately describe small-molecule ligand geometry. However, the fact that the *Mogul* validation results show a statistically significant decrease for *PHENIX*–*AFITT* *versus* *AFITT*–CIF models in angle deviations in contrast to bond lengths is consistent with this interpretation: angles are more susceptible to the nonbonded interactions more appropriately modeled by force-field terms than bonds. While not part of the current study, it is suspected that more complex components of conformation such as torsion angles and non­bonded inter­actions are unlikely to be adequately described under the current paradigm. More importantly, this study has shown that the use of a high-quality small-molecule force field eliminates the need, in a large part, for the user to be either an expert in writing and/or the need to be concerned about the quality of the parameters present in the small-molecule CIF dictionary. Unfortunately, CIF-like restraints dictionaries are in widespread use today because they represent the same geometry restraints function as has been found to function well with protein residues. While *PDB_Redo* (Cereto-Massagué *et al.*, 2013 ▸) has shown that improvements to the quality of the model can be made, we have shown that there is a limit, when using restraint dictionaries, to how much of an improvement can be made to ligand conformation energy and geometry. As a move is made to obtain more accurate models, it is our hope that refinement target functions will more often be implemented according to the paradigm presented here (Borbulevych, Plumley *et al.*, 2014 ▸; Smart *et al.*, 2010 ▸, 2014 ▸) so as to more accurately represent the complex conformational space of small-molecule ligands.
